# Imaging of photoacoustic-mediated permeabilization of giant unilamellar vesicles (GUVs)

**DOI:** 10.1038/s41598-021-82140-4

**Published:** 2021-02-02

**Authors:** Diogo A. Pereira, Alexandre D. Silva, Patricia A. T. Martins, Ana P. Piedade, Dmitro Martynowych, David Veysset, Maria João Moreno, Carlos Serpa, Keith A. Nelson, Luis G. Arnaut

**Affiliations:** 1 CQC, Department of Chemistry, University of Coimbra, 3004-535 Coimbra, Portugal; 2 CEMMPRE-Department of Mechanical Engineering, University of Coimbra, 3030-788 Coimbra, Portugal; 3 Department of Chemistry, Massachusetts Institute of Technology, Cambridge, MA 02139 USA; 4 Institute for Soldier Nanotechnology, Massachusetts Institute of Technology, Cambridge, MA 02139 USA; 5 Hansen Experimental Physics Laboratory, Stanford University, Stanford, CA 94305 USA

**Keywords:** Biomedical engineering, Optical materials, Self-assembly, Carbon nanotubes and fullerenes, Imaging and sensing

## Abstract

Target delivery of large foreign materials to cells requires transient permeabilization of the cell membrane without toxicity. Giant unilamellar vesicles (GUVs) mimic the phospholipid bilayer of the cell membrane and are also useful drug delivery vehicles. Controlled increase of the permeability of GUVs is a delicate balance between sufficient perturbation for the delivery of the GUV contents and damage to the vesicles. Here we show that photoacoustic waves can promote the release of FITC-dextran or GFP from GUVs without damage. Real-time interferometric imaging offers the first movies of photoacoustic wave propagation and interaction with GUVs. The photoacoustic waves are seen as mostly compressive half-cycle pulses with peak pressures of ~ 1 MPa and spatial extent FWHM ~ 36 µm. At a repetition rate of 10 Hz, they enable the release of 25% of the FITC-dextran content of GUVs in 15 min. Such photoacoustic waves may enable non-invasive targeted release of GUVs and cell transfection over large volumes of tissues in just a few minutes.

Giant unilamellar vesicles (GUVs), consisting of extremely thin (~ 4 nm) phospholipid bilayer membranes encapsulating aqueous domains of 1 to 100 µm diameter, mimic important aspects of cellular membrane permeability^1,2^ and are employed in the design of artificial cells^3–5^. GUVs are also prominent examples of drug carriers^6^ and bio-microreactors^7^, and play an important role in the study of membrane biophysics^1,8^. In addition to their appeal as biomimetic models of cells and their useful applications, GUVs also offer opportunities for encapsulation of hydrophilic species in the aqueous pool and hydrophobic molecules in the lipid bilayer as well as visualization of all components with optical techniques.

Controlling the permeability of GUVs by non-contact physical processes allows for non-invasive loading and unloading of macromolecules, and has far-reaching implications in diverse fields such as drug delivery^9^, gene transfection^10^, and synthetic biology^11^. Recent efforts to control the permeability of GUVs have focused on ultrasound^12,13^, alternating magnetic fields^14^, electropulsation^15,16^, and gigahertz acoustic streaming^17^, which are methods that only produce a perturbation when they are turned on, as opposed to chemical methods that change the composition of the organism. These studies have contributed to the understanding of transient permeability of GUVs and biological membranes, but there is still a need for non-invasive physical methods that act over a depth of several centimeters, without cytotoxicity or added facilitating agents. Recently, generation of broadband, high-intensity photoacoustic waves by the thermoelastic effect has been demonstrated in biocompatible materials which, when excited by short laser pulses (duration *τ*_L_ < 10 ns) at moderate laser fluences (*E*_L_ < 100 mJ/cm^2^) launch planar ultrasound pulses with peak pressures *p*_max_ > 5 MPa^18^. These “piezophotonic biomaterials” (light-to-pressure transducers) offer new opportunities for the permeabilization of phospholipid bilayer membranes^10,19^.

The ultrasound pulses generated via the photoacoustic effect have properties that are substantially different from those of conventional ultrasound^20^. In the photoacoustic effect, the pulsed optical energy absorbed by a chromophore is rapidly converted into thermal energy. When the optical heating of the irradiated volume is faster than its thermal expansion and faster than its cooling through thermal transport, the thermoelastic expansion of the heated volume launches a pressure wave. This can be achieved using nanosecond pulsed lasers which can generate pressure waves that are broadband, high-frequency ultrasound pulses. The optical generation of ultrasound pulses using the photoacoustic effect with nanosecond lasers at low laser fluences has been used in photoacoustic calorimetry^21,22^, characterization of materials^23,24^, gene transfection^10^, many imaging and diagnostic techniques^18,25–28^, and even the aesthetics market^29^. The photoacoustic effect is also currently explored in therapy, where administration of a dye or a nanoparticle with some affinity to target tissues is followed by pulsed laser excitation. This differs from the applications described in this work, which are directed to extracorporeal generation of photoacoustic waves.

Piezophotonic biomaterials typically consist of a chromophore with very high absorption coefficient at the laser wavelength, *μ*_a_, embedded in a material with a large coefficient of thermal expansion, *β* (or, more precisely, a large Grüneisen parameter Γ = *c*_s_^2^*β*/*C*_p_, where *c*_s_ is the speed of sound and *C*_p_ is the specific heat capacity at constant pressure). Such materials can absorb virtually all incident laser light within the first few microns. A large amount of thermal energy rapidly deposited in a small volume of a piezophotonic biomatertial leads to photoacoustic waves with peak compressive pressures that may reach *p*_max_ = 12 MPa for planar films^30^. It is well-known that when such materials are rigidly confined, the photoacoustic waves become mostly compressive waves^9,31,32^. The tensile component of these waves is relatively small and the low peak rarefaction pressure leads to low values of the Mechanical Index (MI) even for *p*_max_ in the tens of MPa. This can avoid damage to cells and tissues arising from cavitation when MI > 0.7, because MI is calculated as the ratio between the peak rarefaction pressure (in MHz) to the square root of centre frequency (in MHz)^33^. The success of carbon nanotubes (CNT) or carbon nanofibers with high *μ*_a_ permeated with polydimethylsiloxane (PDMS) with high *β* (or Γ) in generating intense photoacoustic waves led to many efforts to improve the fabrication of CNT-PDMS piezophotonic biomaterials^18,25^.

The incorporation of fluorophores inside GUVs or in their phospholipid bilayers enables detailed observations of the impact of photoacoustic waves in GUVs using confocal microscopy. However, the real-time visualization of photoacoustic waves and GUVs with micrometer precision is crucial to understand membrane permeability. A variety of imaging methods including interferometric imaging techniques have allowed for direct visualization of laser-driven acoustic and shock waves^34–36^. In this work, we use ultrafast interferometric techniques coupled with *in-situ* fluorescence imaging to visualize the permeabilization of fluorescence-labelled GUVs by highly compressive (*p*_max_ > 1 MPa) photoacoustic waves generated with piezophotonic films at moderate laser fluences (*E*_L_ ≤ 100 mJ/cm^2^). We show that the photoacoustic waves enable large molecules and proteins to cross the phospholipid bilayer without damaging GUVs. GUV integrity and extent of permeabilization are monitored by confocal microscopy and flow cytometry. Interferometric images, collected at selected time delays between the laser pulse that generates photoacoustic waves and the probe laser pulse, capture the propagation of the photoacoustic waves toward and through the GUVs. Fluorescence measurements show that repeated exposure of the GUVs to the photoacoustic waves can increase the membrane permeability while preserving membrane integrity.

## Optical-to-pressure transduction

**Figure 1:**
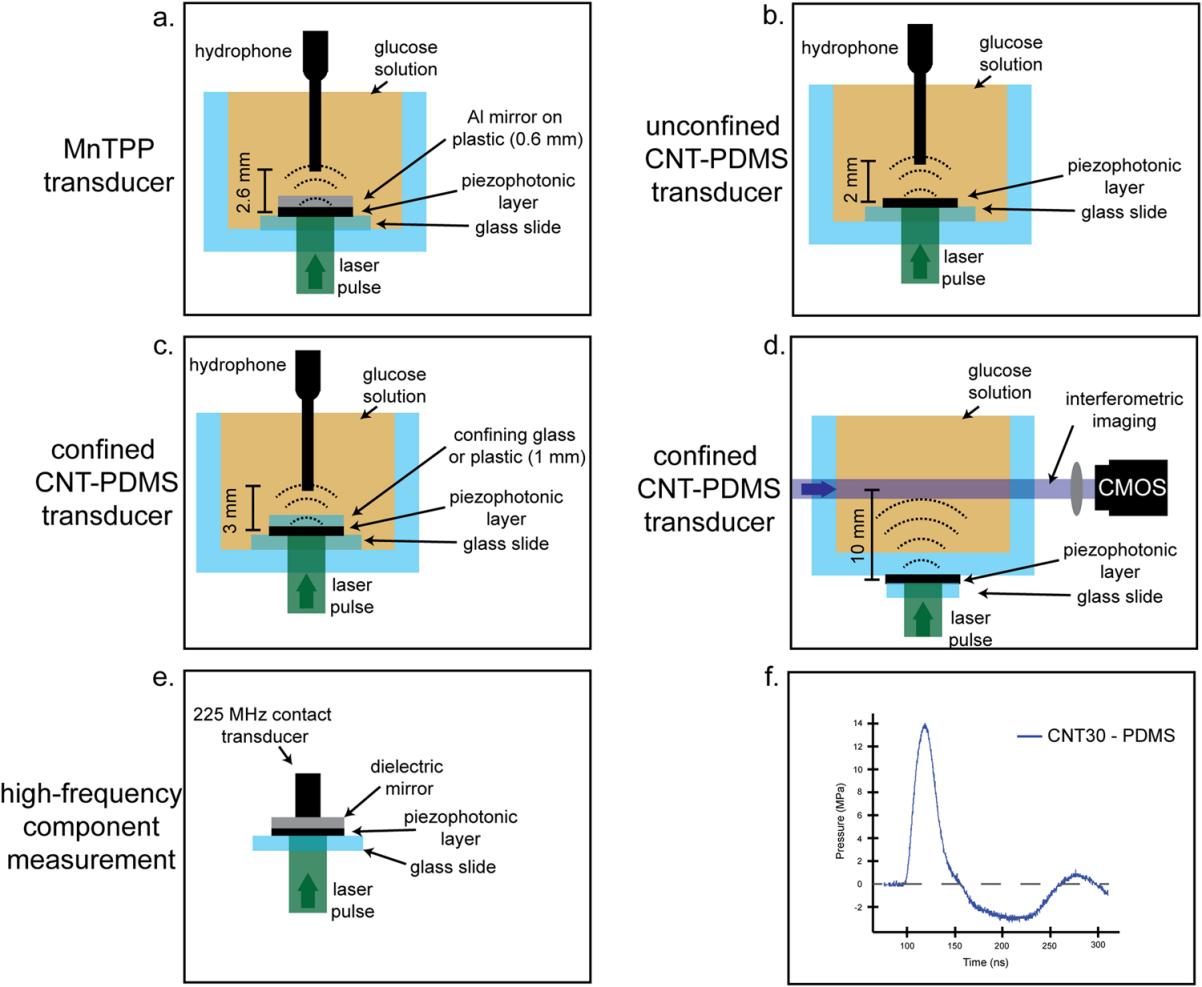
Configurations employed to measure absolute pressures with hydrophone and frequency distributions with contact transducer. (**a**) MnTPP deposited on a glass slide and confined with an aluminium mirrored plastic cover. (**b**) CNTs grown on a glass slide and infused with PDMS. (**c**) Similar CNTs infused with PDMS but now covered with either a plastic cover or a glass slide. (These samples are referred to “CNTxx-PDMS-plastic” or “CNTxx-PDMS-glass” in the text, where “xx” indicates the CNT thickness in microns.) (**d**) Similar CNTs infused with PDMS but now pressed against the side of a cuvette. (**e**) Measurement of photoacoustic waves with a 225 MHz contact transducer. (**f**) Example of a photoacoustic wave generated with unconfined CNT30-PDMS absorbing a 100 mJ/cm^2^ laser pulse, and measured with a calibrated 30 MHz hydrophone.

The permeabilization of a large number of GUVs, or cells, without damage requires the repeated generation of mostly compressive ultrasound pulses with high peak pressures propagating as planar waves over a large volume. We employed polystyrene films doped with manganese (III) chloride 5, 10, 15, 20-tetraphenylporphyrinate (MnTPP) and deposited on glass slides, described in earlier work^10^, to generate such ultrasound pulses. For higher intensity ultrasound pulses we employed CNT grown to different thicknesses over glass slides and infused with PDMS. We employed 8-ns, 532-nm laser pulses (see “Methods” section for details) to generate photoacoustic waves in these materials and measured the waves with a needle hydrophone, a 225-MHz transducer, or an interferometer. Figure 1 shows the types of measurements made. CNT films without PDMS did not give measurable signals. This was expected as CNTs have small, or even negative, coefficients of thermal expansion. However, they have very large thermal conductivities which allows for fast heat transfer to PDMS, which undergoes thermoelastic expansion and launches the photoacoustic wave. Figure 1f shows the characteristic time profile of a photoacoustic wave, with a compressional peak pressure one order of magnitude higher that the rarefaction tail and a FWHM of 25 ns, which give pressure gradients of 0.5 GPa/µm, difficult to produce with conventional ultrasound sources.

**Figure 2:**
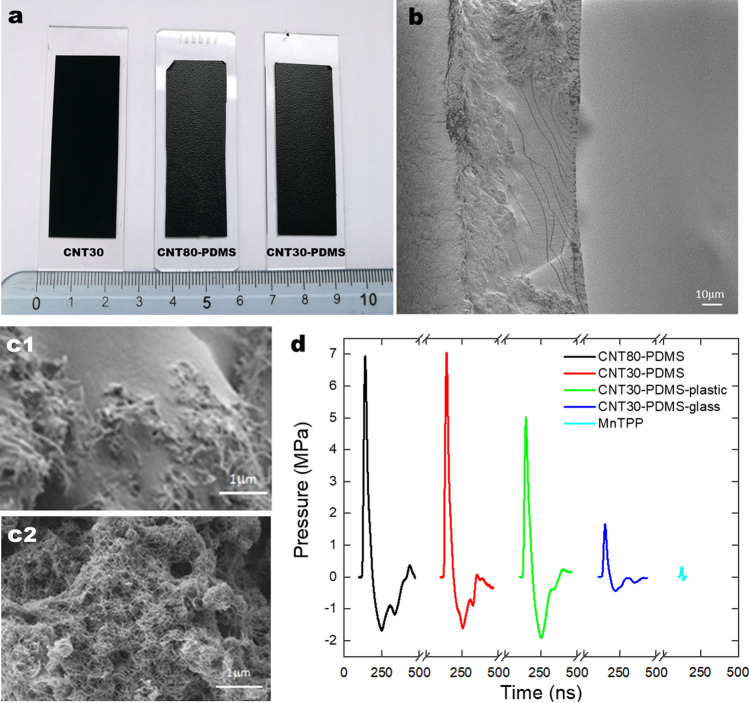
CNT-PMDS films. (**a**) Photographs of the films without PMDS (CNT30) or infused with PDMS and different thicknesses (CNT80-PDMS, CNT30-PDMS). (**b**) SEM micrograph of the cross section of CNT80-PDMS. The free surface is on the right and the interface with the glass slide on the left. (**c**) SEM micrographs of the surfaces of CNT80-PDMS (**c1**) and of CNT30 (**c2**). (**d**) Photoacoustic waves measured with hydrophone (arbitrary origins displaced in the x-axis for clarity of presentation of the acoustic waves, which have essentially the same travelling time between source and detector), obtained with laser pulses of 50 mJ/cm^2^ at 532 nm; the temporal FWHM of the compressive wave generated by CNT30-PDMS is 25 ns.

Figure 2 shows CNT films and typical pressure waves. Although the thicknesses of the nanotube layers are not homogeneous in each material, they have thicknesses of ~ 80 µm (CNT80-PDMS) and ~ 30 µm (CNT30-PDMS). Relevant properties for the generation of photoacoustic waves are collected in Table 1. Absorption spectra are shown in Supplementary Fig. S1. Higher frequency components of the photoacoustic waves were assessed with a 225 MHz transducer as shown in Fig. 1 and are presented in Supplementary Fig. S2.

**Table 1:** Properties of piezophotonic films.

Film	Thickness, *l* (µm)	Absorbance, *A*	*µ*_a_ (cm^–1^)^a^	*p*_max_ (MPa)
CNT30	30	> 10	> 7600	~ 0
CNT80-PDMS	80	> 10	> 2800	6.9
CNT30-PDMS	30	> 10	> 7600	7.0
CNT30-PDMS-plastic	30	> 10	> 7600	5.0
CNT30-PDMS-glass	30	> 10	> 7600	1.7
MnTPP	70	1.23	400	0.32

At 532 nm for a laser fluence of 50 mJ/cm^2^.

^a^Napierian absorption coefficient, *µ*_a_ = 2.3 *A*/*l.*

The CNT30-PDMS-plastic configuration is comparable to that of MnTPP, which was previously employed in gene transfection^10^. CNT30-PDMS-glass is similar to the sample configuration used for interferometric experiments. The comparison between CNT30-PDMS-plastic and MnTPP reveals that CNT impregnated with PDMS generates photoacoustic waves 16 times greater in amplitude than MnTPP in polystyrene. The lower peak pressure of CNT30-PDMS-glass *vs*. CNT30-PDMS-plastic, 1.7 *vs*. 5.0 MPa, can be related by the difference in acoustic impedance (*Z*) between glass or plastic and water. This was compensated in interferometric measurements by increasing the laser fluence to 100 mJ/cm^2^. The highest pressure measured was *p*_max_ = 14.1 MPa for CNT30-PDMS at 100 mJ/cm^2^ using 1064 nm laser pulses (Fig. 1f). We used the fundamental rather than the second harmonic (532 nm) of the Nd:YAG laser in this experiment to have more energy per pulse. This value may be slightly underestimated because the FFT of the waves measured with the 225-MHz transducer reveal frequency components that extend beyond the calibration range of the hydrophone (1–30 MHz). This film is in direct contact with water and does not suffer the impedance mismatch. Photoacoustic waves were stable and reproducible for at least 30 min at a 10 Hz laser pumping frequency for laser fluences up to 100 mJ/cm^2^. CNT films with no PMDS degraded after a single laser shot at this fluence, suggesting that without thermal energy dissipation into PDMS, the CNT films degrade very quickly.

The loss in intensity of photoacoustic waves crossing the glass-solution interface motivated the fabrication of piezophotonic biomaterials with higher optical-to-pressure transduction than MnTPP films to enable proper observation of GUV permeabilization using interferometry. The CNT films employed in this work are advantageous because of their exceptionally high absorption coefficients. When optical energy is converted into thermal energy in smaller volumes, higher intensity pressure waves are generated. CNT30-PDMS-plastic and MnTPP in Table 1 refer to hydrophone measurements of photoacoustic waves that crossed similar interfaces. The order of magnitude higher *p*_max_ of CNT30-PDMS-plastic is largely due to its higher *µ*_a_.

Quantitatively^25^, for laser pulses with *τ*_L_ = 8 ns, CNT30-PDMS has (*µ*_a_)^–1^ << *c*_s_*τ*_L_ and is classified as a “thin absorber” characterized by

1 $$  \rho _{{\max }}  = \Gamma E_{{\text{L}}} /c_{{\text{s}}} \tau _{{\text{L}}}   $$

whereas MnTPP is in the limit of a “thick absorber”, (*µ*_a_)^−1^ > *c*_s_*τ*_L_, where

2 $$  \rho _{{\max }}  = \Gamma E_{{\text{L}}} \mu _{{\text{a}}}   $$

The expressions above assume that all optical energy absorbed was rapidly transformed into heat. Decreasing the thickness of a thin absorber does not have an important impact on the peak pressure.

## Real-time interferometric imaging

**Figure 3:**
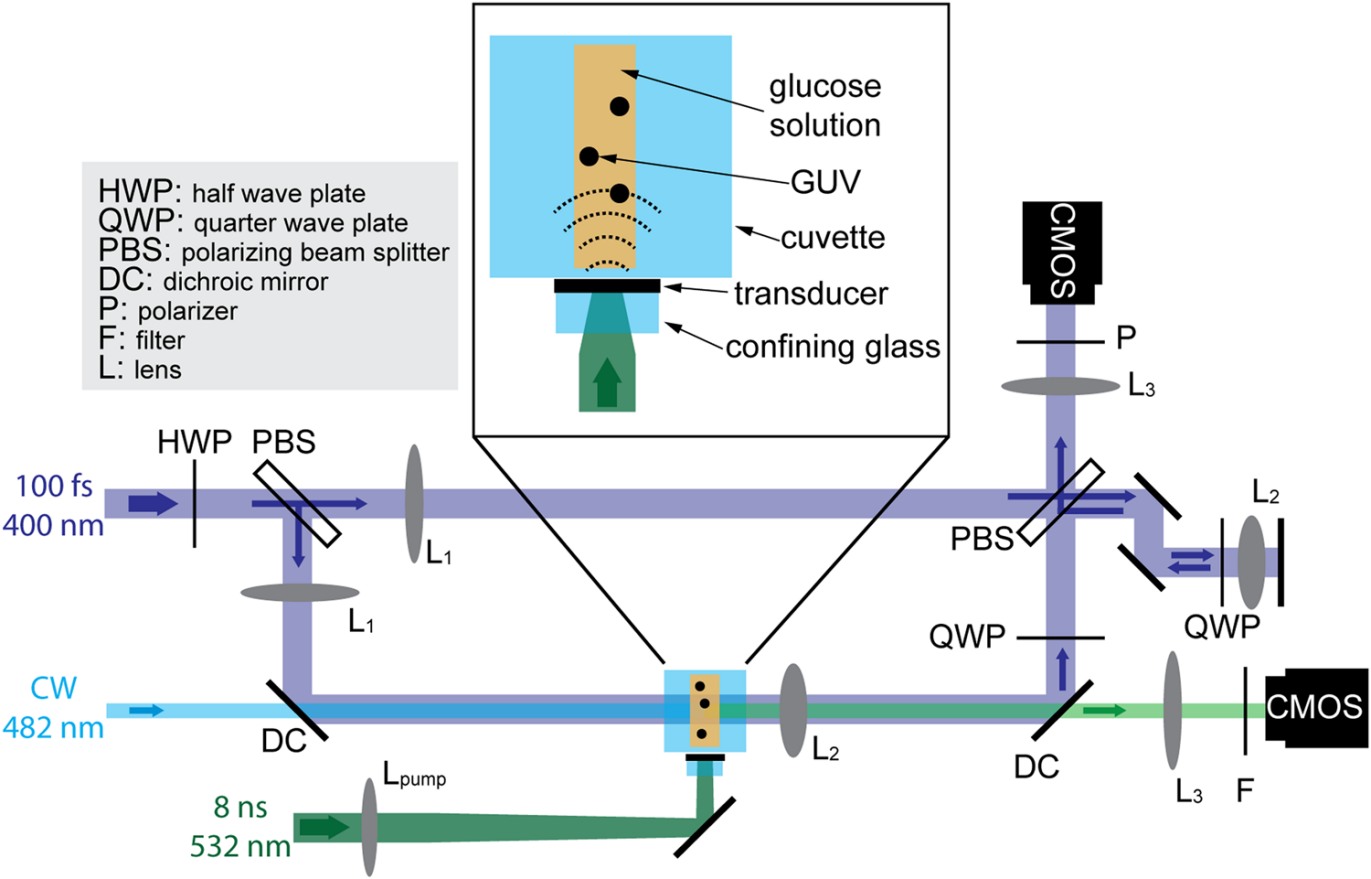
Apparatus for analyzing photoacoustic waves interacting with GUVs. Nd:YAG 532-nm pump laser pulses are totally absorbed by the piezophotonic transducer that generates ultrasound pulses. A frequency-doubled Ti:sapphire femtosecond laser pulse is split in reference and sample arms and their interference is recorded by a camera. Another camera is used to detect the fluorescence of FITC-dextran resulting from excitation by a CW laser. The zoom of the 1.5 × 1.5 cm polished quartz cuvette shows details of the assembly of the transducer film. The observation window is 7 to 9 mm from the plane of the transducer film.

**Figure 4:**
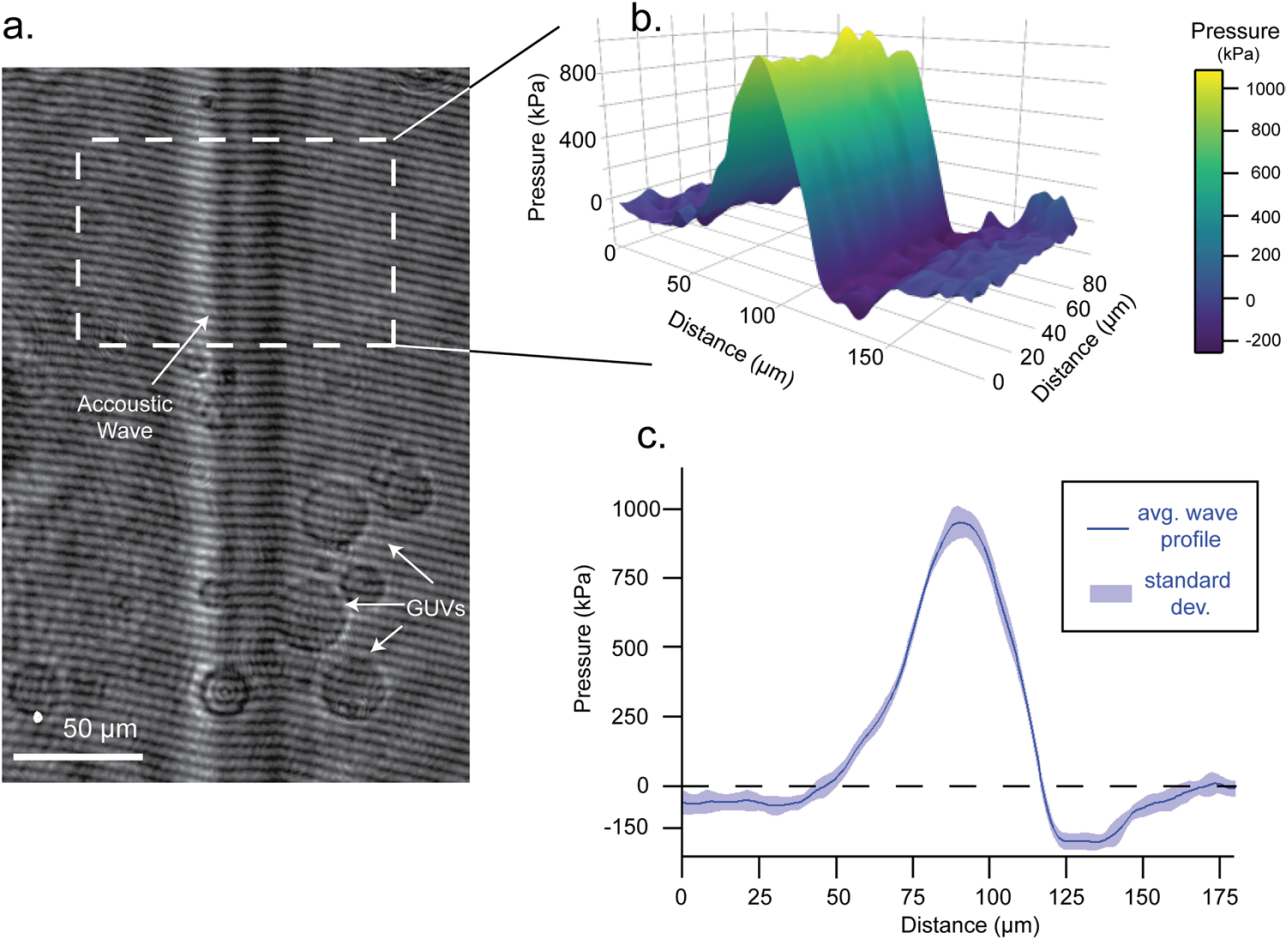
Interferometric images and photoacoustic waves. (**a**) Interferometric image of a photoacoustic wave generated with a CNT30-PDMS film pressed against the cuvette and a laser fluence of 100 mJ/cm^2^. (**b**) Map of pressure distribution extracted from the interferogram in the region of interest. (**c**) Average pressure profile in the shorter dimension as well as the standard deviation from this profile.

Imaging the propagation of the acoustic waves in solution and their interaction with GUVs was achieved with the setup presented in Fig. 3, which was adapted from earlier work^35^. We acquired interferometric images with a Mach–Zehnder interferometer and a variably-delayed probe laser pulse. These interferometric images allowed for direct visualization of the acoustic wave, manifesting as bends in the fringes of the interferometric image (Fig. 4). Synchronizing the laser producing the acoustic waves with the imaging laser and varying the delay between the lasers allowed for time resolved movies to be built up showing the wave propagation and interaction with the GUVs.

The acoustic impedance mismatch between the cuvette and the solution lowered the transmission of the photoacoustic wave. The intensity transmission coefficient

3 $$  T = 4Z_{1} Z_{2} /(Z_{1}  + Z_{2} )^{2}   $$

from the material 1 to water (impedance *Z*_2_ = 1.494 × 10^6^ N s m^–3^) ranges from *T* = 0.97 for PDMS (*Z*_1_ = 1.048 × 10^6^ N s m^–3^)^37^, to *T* = 0.94 for polystyrene (*Z*_1_ ≈ 2.5 × 10^6^ N s m^–3^) and *T* = 0.35 for glass (*Z*_1_ ≈ 14 × 10^6^ N s m^–3^). We employed CNT30-PDMS films pressed against the cuvette (Fig. 1d) and used a laser fluence of 100 mJ/cm^2^ to obtain high compressive pressures and visualize their interaction with GUVs under conditions where GUVs are permeabilized and release large hydrophilic molecules.

## Interferometric and fluorescence analysis

Interferometric images were collected with and without the acoustic pump laser on. Using a 2D Fourier transform method, these images were converted to 2D phase images, using a Hahn function for apodization of the spectrum^38,39^. Subtraction of phase images with and without acoustic pumping yields the phase change caused by the acoustic wave, Δ*φ.* This phase change can be used in Eq. (2) to calculate the change in the refractive index Δ*n*

4 $$  \Delta n = \frac{{\Delta \varphi }}{{2\pi }}\frac{\lambda }{l}  $$

where *l* is the thickness of the liquid layer (2 mm) and *λ* is the wavelength of light (400 nm). This change in refractive index is then used to determine the density change Δ*p* by the empirically derived equation^40^

5 $$  \Delta \rho  = \frac{{\Delta n}}{{0.322}}\,[{\text{g}}/{\text{cm}}^{3} ]  $$

Finally, the pressure *P* is calculated using the Tait equation of state

6 $$P= {k}_{0}/n\left[(\rho/{\rho }_{0})^{n}-1\right]+{P}_{0}$$

Pressure maps derived from this analysis describe the wave structure and are presented in Fig. 4. The peak pressure measured with this procedure 10 mm from the film, ~ 1 MPa, is consistent with the design of CNT30-PDMS films with glass interfaces, which give *p*_max_ = 2.4 MPa at 100 mJ/cm^2^ pump fluence according to hydrophone measurements 3 mm from the film and 1.5 MPa at 10 mm (see Supplementary Table S1 for the distance dependence of the peak pressure). The spatial extent (FWHM) of the photoacoustic wave in Fig. 4 is ~ 36 µm, which is approximately the diameter of GUVs and of many eukaryotic cells, and is consistent with the result of hydrophone data in Fig. 2d for CNT30-PDMS films which showed a temporal FWHM of 25 ns.

Pressure gradients can exert mechanical forces and perturb structures that are commensurate with their widths. A possible outcome of this perturbation is the permeabilization of the phospholipid bilayer and the leakage of their content. This was investigated with fluorescent-labelled GUVs.

## Permeabilization of GUVs

**Figure 5:**
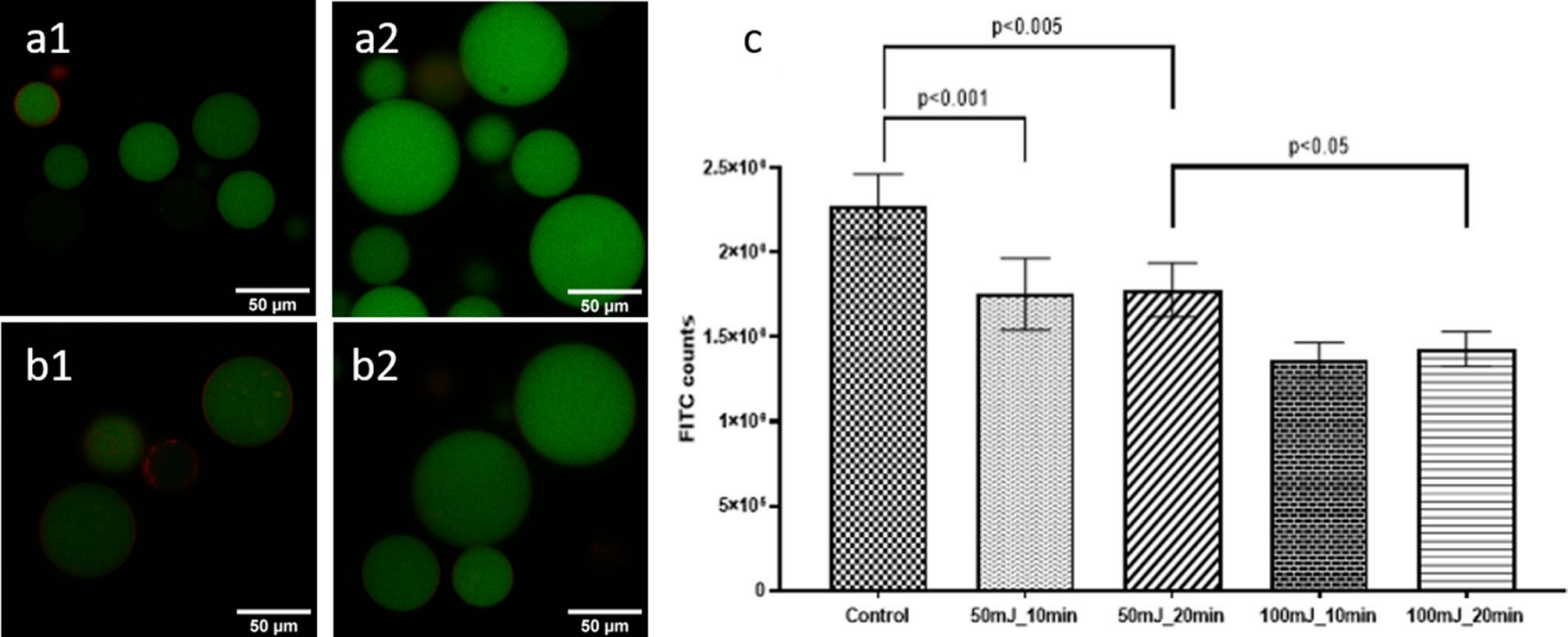
Confocal images and flow cytometry of GUVs containing FITC-dextran. (**a**) Representative examples of images of GUVs before exposure to photoacoustic waves, and (**b**) after exposure to photoacoustic waves (**b1**) exposure to 50 mJ/cm^2^ for 20 min; (**b2**) exposure to 100 mJ/cm^2^ for 10 min). (**c**) Flow cytometry counts of fluorescence intensity inside the GUVs before and after exposure to photoacoustic waves. Uncertainties in the results are indicated by error bars.

GUVs were prepared with 1-palmitoyl-2-oleoyl-*sn*-glycero-3-phospacholine (POPC) containing 1 mol% 1,2-dioleoyl-*sn*-glycero-3-phosphoethanolamine-N-(lissamine rhodamine B sulfonyl) (Rho-DPPE). Rho-DPPE locates in the phospholipid bilayer and emits fluoresce in the red when excited at 561 nm. Sucrose solutions containing fluorescein isothiocyanate-dextran (FITC-dextran, 4,000 Da, hydrodynamic radius *r*_h_ = 1.5 nm) or Green Fluorescent Protein (GFP, 26,900 Da, *r*_h_ = 2.5 nm)^41^ were employed to hydrate the lipid films. Either FITC-dextran or GFP remain contained inside the GUVs and emit green fluorescence when excited at 488 nm. Figure 5 presents representative confocal images of the GUVs with the membrane labelled in red with Rho-DPPE and containing FITC-dextran.

**Figure 6:**
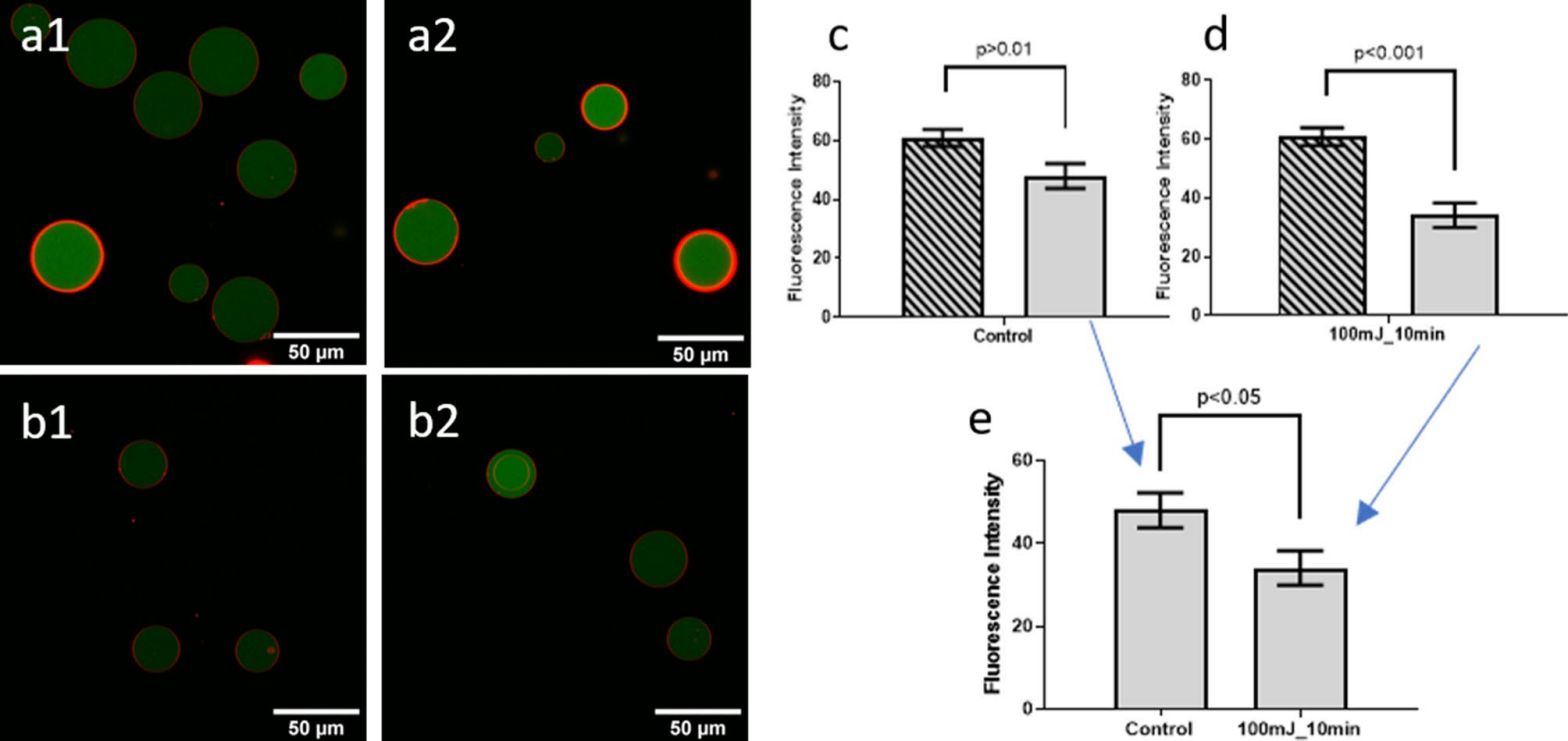
Confocal images of GUVs containing GFP and image analysis. (**a**) Representative examples of images of GUVs before exposure to photoacoustic waves, and (**b**) after exposure to photoacoustic waves generated with laser pulses at 100 mJ/cm^2^ for 10 min. (**c**) Control before and after all procedures except exposure to photoacoustic waves, to assess the effect of manipulating GUVs for 2 h. (**d**) Fluorescence before and after exposure to photoacoustic waves. (**e**) Comparison between control GUVs after the procedures of exposure, but without exposure to photoacoustic waves, and GUVs exposed to photoacoustic waves.

GUVs containing FITC-dextran were exposed to photoacoustic waves generated with MnTPP films for 10 or 20 min, either at 50 or 100 mJ/cm^2^ pump fluence. Changes in area and in fluorescence intensity inside the GUVs were followed by confocal microscopy and by flow cytometry. Figure 5 shows the analysis of the fluorescence by flow cytometry. Supplementary Fig. S3 presents flow cytometry data. There are statistically significant fluorescence decreases after exposure to photoacoustic waves generated with 50 mJ/cm^2^ laser pulses (*p* < 0.005), as well as between the GUVs exposed to 50 and 100 mJ/cm^2^ (*p* < 0.05). Figure 5 also shows representative confocal microscopy images of GUVs exposed in two experimental conditions. Supplementary Fig. S4 shows the statistical analysis carried over all the images. These independent experiments confirm the significant (*p* ≤ 0.01) release of FITC-dextran by the GUVs when subject to photoacoustic waves. Figure 6 shows similar experiments for GUVs containing GFP. The decrease of GFP fluorescence inside the GUVs after exposure to photoacoustic waves is comparable to that observed with FITC-dextran.

The size of GUVs before and after the exposure to photoacoustic waves was evaluated by confocal microscopy and possible changes in the number of GUVs were evaluated by flow cytometry. Supplementary Figures S5 and S6 show that neither the area nor the number of GUVs changed significantly with the exposure to photoacoustic waves. Hence, exposing the GUVs for 10–20 min to photoacoustic waves generated with 100 mJ/cm^2^ decreases the fluorescence intensity of FITC-dextran by 32% without destroying the GUVs or changing their sizes.

## Real-time imaging of GUVs permeabilization

**Figure 7:**
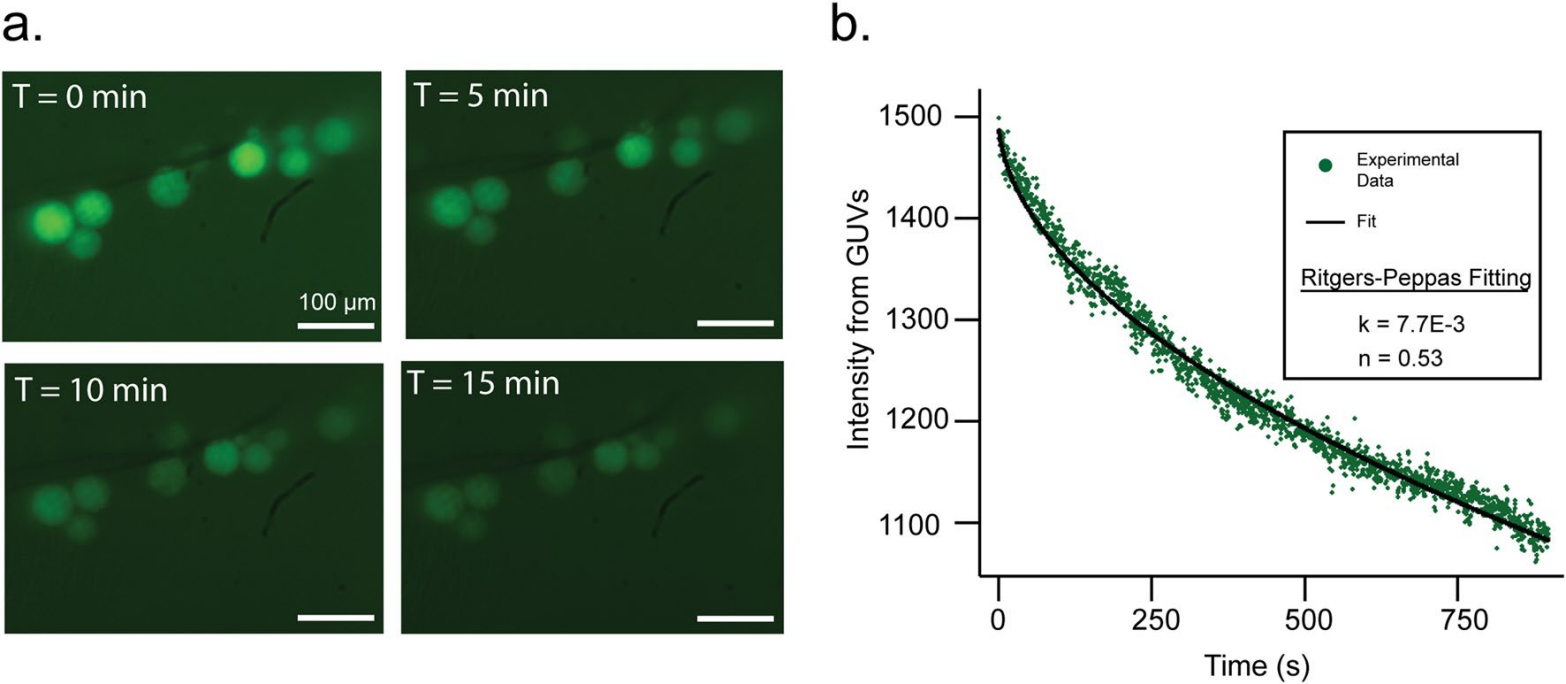
Real-time confocal images and analysis. (**a**) Fluorescence of FITC-dextran at different times of exposure to photoacoustic waves generated with a 10-Hz frequency from stationary GUVs, using CNT30-PDMS films. (**b**) Fluorescence intensity decrease from fits with the Ritger-Peppas equation for solute release, taking (1 − *I*_t_/*I*_0_) as the fraction of drug release, where *I*_t_ is the fluorescence at time *t* and *I*_0_ the initial fluorescence, both corrected for background fluorescence. The laser fluence at the piezophotonic layer was 100 mJ/cm^2^, but interferograms show that the GUVs were exposed to ~ 1 MPa pressure pulses.

Real-time fluorescence imaging of the GUVs under acoustic pumping was performed as shown in Fig. 3. Figure 7 shows time-resolved changes in fluorescence of stationary GUVs. These changes are consistent with the results from confocal microscopy and flow cytometry and enable the measurement of GUV release kinetics. We characterize this release with the Ritger–Peppas equation^42^

7 $$  M_{{\text{t}}} /M_{\infty }  = kt^{n} \,{\text{for}}\,M_{{\text{t}}} /M_{\infty }  < 0.6  $$

where *M*_t_/*M*_∞_ is the drug fraction released at time *t* with kinetic constant *k* and *n* is a diffusion exponent related to the drug transport mechanism. Ritger and Peppas showed that for a spherical device, such as a GUV, Fickian diffusion and zero-order release yield *n* = 0.43 and 1.0, respectively. We do not expect Fickian diffusion because we have on–off perturbations of the bilayer membrane with 10 Hz frequency, possibly with membrane resealing in between perturbations. Time-independent release of FITC-dextran is not expected either because it would likely mean erosion of the GUVs. Fitting the data of Fig. 7 under the assumption that the fluorescence intensity is zero at *t* = ∞ gives *n* = 0.53.

## Discussion

Earlier permeabilization studies of biological membranes with photoacoustic waves employed MnTPP films that generated peak pressures ~ 1 MPa^9,10^. These photoacoustic waves were not cytotoxic and in 10 min enabled 5% transfection of plasmid DNA encoding GFP in COS-7 monkey fibroblast cells. Cell transfection with intense shock waves (> 30 MPa) produced by laser ablation of a target material showed lower efficacy and higher cytotoxicity^43,44^. Nevertheless, 5% efficiency is low relative to the efficiencies of established physical transfection methods such as electroporation^45^. The absence of cytotoxicity in transfection with photoacoustic waves leaves space for improvement, however its full exploitation needs information on the mechanism of cellular membrane permeabilization.

The permeabilization of GUVs with photoacoustic waves generated by MnTPP films in this work is similar to that of transfection experiments^10^. The waves generated in MnTPP propagated through the plastic cover of MnTPP and through the bottom of the recipient containing the glucose solution before reaching the GUVs. The peak pressure generated with MnTPP films with plastic cover is 0.3 MPa for a laser fluence of 50 mJ/cm^2^ but the peak pressure measured in solution is 2/3 of this value due to the additional interface crossed. Hence, GUVs in the bottom of the well are subject to *p*_max_ ≈ 0.2 or 0.4 MPa for laser fluences of 50 or 100 mJ/cm^2^, respectively. In the transfection experiments, the monolayer of cells adhered to the bottom of the well was exposed to *p*_max_ ≈ 0.68 MPa^10^.

We ensured that photoacoustic waves did not destroy GUVs or reduce their sizes (Supplementary Figs. S5 and S6). While the GUVs did not change in size or in number, Fig. 5 show that they released FITC-dextran, especially during the first 10 min of exposure to higher intensity photoacoustic waves. The analysis of confocal images such as those in Fig. 5 confirms these results (Supplementary Fig. S3). The release of GFP in the same conditions (Fig. 6) further demonstrates the ability of photoacoustic waves to permeabilize phospholipid bilayer membranes while preserving their integrity.

It is notable that CNT30-PDMS can generate planar photoacoustic waves with *p*_max_ = 14.1 MPa at laser fluences of 100 mJ/cm^2^ for tens of thousands of laser pulses. These are among the largest peak pressures of planar ultrasound waves launched by thermoelastic expansion of piezophotonic biomaterials into aqueous media that have been measured millimetres away from the source^18,25^.

Analysis of the interferometric images yielded clear spatial profiles of the acoustic waves. Peak pressures were consistent shot-to-shot and stable across the entire image field of view (~ 800 µm acoustic propagation length). The wave structure in Fig. 4 is representative of the waves produced, exhibiting a steep rise and little to no rarefaction. Peak pressures expected from hydrophone measurements for 100 mJ/cm^2^ laser pulses (2.9 MPa) are consistent with the pressures deduced from interferometric imaging (~ 1 MPa) in view of the differences in the equipment employed and distance from the source (Supplementary Table S1). Photoacoustic waves are mostly compressive in both cases. The small rarefaction tail is unlikely to produce cavitation, which would be readily observable in the optical images if it occurred^46^. Their FWHM values are also very similar although they were measured at very different distances from the source. An important difference between shock waves and photoacoustic waves is that only approximately 10% of the acoustic energy generated by a shock wave reaches a distance of 10 mm^47^, and most of this energy is converted into heat in first 0.2 mm of shock front propagation, whereas photoacoustic waves travel as ultrasound pulses^20^. This explains the release kinetics of GUVs located at least 7 mm from the acoustic source.

Considering the hydrodynamic radii of FITC-dextran and GFP, exposure to photoacoustic waves must open transient pores in their membranes with diameters of at least 5 nm. An upper limit was not investigated in this study, but earlier work on the transfection of plasmid DNA encoding GFP (gWizGFP, 3.74 MDa), which is estimated to have a radius of gyration of 600 nm^2^ and, consequently^48^, a hydrodynamic radius of 400 nm, suggests that photoacoustic waves may open transient pores with diameters approaching 1 µm. GHz acoustic streaming generates shear stress that induces the generation of pores with ca. 100 nm diameter in polymer-shelled vesicles without destroying them^17^. Electroporation of GUVs forms long-lived (~ 10 ms) macropores of micrometre size, which are decisive for increase in permeability, but lead to lipid loss and decrease in GUV size^2,15,16^. The macropores of electroporation are observed at GUV poles when deformed to ratios of perpendicular axes (aspect ratios) of 1.5 under square-wave pulses of 3 kV/cm for 100 µs^15^. The GUV aspect ratio upon stimulation by acoustic streaming is 1.2^17^. The change in GUV shape induced by each photoacoustic wave is expected to be even more subtle, but exposing GUVs containing FITC-dextran to 9,000 waves leads to a 25% decrease in FITC-dextran content, as shown in Fig. 7.

The GUV release kinetics, with a diffusion exponent of *n* = 0.53, are comparable with magnetically triggered release of drugs from magnetoliposomes perturbed by magnetic fields, and are consistent with the formation of pores in the membrane^49^.

## Outlook

We used nanosecond laser pulses to generate mostly-compressive planar photoacoustic waves with peak pressures up to 14 MPa using carbon nanotubes infiltrated with polydimethylsiloxane. Waves of a few MPa launched by the thermoelastic expansion of the films can be used thousands of times without degradation or damage to phospholipid bilayer membranes. Under acoustic pumping, the membranes become transiently permeable to large molecules and proteins. Real-time detection of the interaction between photoacoustic waves and GUVs using interferometric imaging explain their drug release kinetics. Broadband and high-intensity photoacoustic waves offer a very simple tool to permeate cells or vesicles in volumes higher than 10 cm^3^ without toxicity, in 10 min. GUVs are both good models of human cells and effective drug delivery systems. The non-destructive permeabilization of GUVs with photoacoustic waves can be used in targeted drug delivery. The major challenge in such applications is to develop biocompatible GUVs with adequate pharmacokinetics. The permeabilization without damage to the lipid membranes also offers opportunities for gene delivery. Photoacoustic waves have the distinct ability to increase the permeability of billions of cells at the same time without compromising their viability. This is expected to allow for gene transfection of a targeted tissue without local or systemic toxicity.

## Methods

### Giant unilamellar vesicles

GUVs were prepared by the gel-assisted swelling method^50,51^ with some modifications. A volume of 200 μL of polyvinyl alcohol (PVA) 5% (w/w) in 280 mM sucrose, previously heated to 90 °C and allowed to cool to ~ 40 °C, was spin-coated (Speciality Coating System, Inc., Model P6700, 1200 rpm, 120 s) on the wells of an uncoated μ-slide 8-well (Ibidi, Germany) and allowed to dry for 15 min at 50 °C. The μ-slide was then placed on top of a heating plate (~ 40 °C) and 15 μL of a lipid solution in chloroform (POPC 1.5 mM containing 1 mol% Rho-DPPE) were added at the centre of each well. The μ-slide was placed under vacuum for 15 min in a desiccator to remove traces of solvent. The resulting lipid films were hydrated with 300 μL per well of sucrose 280 mM containing FITC-dextran 200 μM or GFP 44 μM and maintained stationary and protected from light for 2 h. Swelling of the lipid film leads to the formation of GUVs (see Supplementary Fig. S7) that are detached from the film by gentle agitation, transferred to Eppendorf tubes and allowed to rest for 30 min in the dark. To remove the dye outside the GUVs, 300 μL of GUVs suspension was gently added to the top of conical bottom glass tubes containing 10 ml glucose 280 mM and the GUVs were allowed to sediment for 1 h in the dark. GUVs containing sucrose have a higher density than glucose solutions and slowly deposit on the bottom of the flask, with the larger GUVs depositing first, which allows for a selection of sizes. The top 10 ml were gently removed after sedimentation and replaced by 10 mL of fresh glucose 280 mM solution. The solution was allowed to rest for 1 h and 300 μL were collected from the bottom of the tube and stored in Eppendorf tubes until further use. This procedure gives a good contrast (fluorescent dye in the outside media diluted ~ 66 times) and a selection of the larger GUV population that affords a more homogeneous sample.

### Piezophotonic biomaterials and photoacoustics

MnTPP was homogeneously incorporated in polystyrene films with thicknesses between 70 and 80 µm following published procedures^9,10^. Vertically-aligned carbon nanotubes (CNT) infused with PDMS were prepared by Surrey NanoSystems Ltd (Newhaven, UK). Absorbances were measured with a Cary 5000 Series UV–Vis–NIR Spectrophotometer (Agilent Technologies). Absolute pressures were measured with a 0.2 mm 30 MHz needle hydrophone (Precision Acoustics model NH0200) in a water pool at 20 °C. The MnTPP film was confined between a 1 mm thick glass window and an aluminum back mirror on a 0.6 mm thick plastic support. CNT-PDMS films deposited on a 1 mm thick glass slide were tested in the configurations shown in Fig. 1. Acoustic couplings were optimized by spreading a very thin layer of silicone on the cover and pressing it against the film. The laser pulses were directed through the glass slides to the films. In MnTPP, CNT30-PDMS-plastic and CNT30-PDMS-glass, the photoacoustic wave generated in the film had to cross the cover before propagating into the water to reach the hydrophone. The hydrophone was placed within 2 mm of the cover of the film in CNT30-PDMS. The volume of the solution was ~ 60 mL. The pressure waves were calibrated according to the calibration data provided by the hydrophone manufacturer for the 1 to 30 MHz range, as discussed in detail in the Supplementary Information. Photoexcitation of the films employed the second harmonic (532 nm) of a nanosecond laser pulse (Quantel Ultra 50 Q-Switched Nd:YAG, 8 ns pulse FWHM, 10 Hz). The PA wave profiles were recorded with a DPO7254 Tektronix digital oscilloscope (2.5 GHz bandwidth). A high frequency (225 MHz) contact transducer from Panametrics/Olympus (model V2113) was also employed to investigate the presence of high frequencies in the photoacoustic waves. These measurements used the front-face irradiation design of photoacoustic calorimetry^21,23^, illustrated in Fig. 1e. The front-face dielectric mirror protects the transducer from laser light and delays the photoacoustic wave generated in the piezophotonic layer to separate it temporally from electronic noise resulting from firing the laser. The glass slide, piezophotonic layer and mirror were pressed against the surface of the contact transducer. All moving parts were acoustically coupled with silicone.

### Confocal fluorescence

Confocal fluorescence microscopy was performed at the MICC Imaging facility of the Centre for Neuroscience and Cell Biology (Coimbra, Portugal) using a Carl Zeiss, LSM 710 microscope. Both for FITC-dextran and GFP, six confocal images were collected for each experimental condition from different regions of the solution using the Zeiss ZEN software. The images were manually selected to obtain a region of interest in a confocal plane with five to fifteen GUVs. The images collected with the ZEN software were studied with the Image J software. Here, each GUV in the region-of-interest was manually selected and information on fluorescence intensity and size was extracted. Three independent experiments were performed with FITC-dextran, each one with more than six confocal images of GUVs. The analysis of the GUVs was performed before and after exposure to the photoacoustic waves. The images before and after exposure are independent. We report two types of controlled experiments. One evaluates the perturbation of GUVs moved between labs, and compares the initial GUVs fluorescence intensity with that obtained after manipulations identical to those of GUVs exposed to photoacoustic waves. The other controlled experiment compares the intensity of the control GUVs after manipulations with the GUVs with the same manipulations plus exposure to the photoacoustic waves.

### Flow cytometry

For quantitative and automated analysis of GUVs, a Cytometer Novocyte 300 was used. Control GUVs and GUVs exposed to photoacoustic waves were placed in a 96 wells microplate and 150 µL of each GUVs sample was analysed. The gating strategy allowed to extract information on the number of GUVs and FITC fluorescence intensity (FITC counts) using a 488-nm excitation laser and an emission filter with a window between 500 and 560 nm. Three independent experiments were performed for each system.

### Scanning electron microscopy

The morphology of the cross-sections and surfaces of CNT films was studied at Instituto Pedro Nunes (Coimbra, Portugal) using a FEG-SEM Zeiss – Merlin/Gemini II equipment.

### Interferometry and imaging

The imaging probe light was provided by an amplified Ti:sapphire laser (800 nm, 100 fs), frequency doubled to 400 nm in a β-barium borate (BBO) crystal. The imaging light was directed into an imaging Mach–Zehnder interferometer. The light was split between sample and reference arms using a polarizing beam splitter, both arms were then telescoped with lens L_1_ (40-cm focal length) and imaged (after the cuvette in the case of the sample arm) with lens L_2_ (3-cm focal length). The pulses were then recombined after the sample in a second polarizing beam splitter, and imaged onto the camera with lens L_3_ (30-cm focal length). The contributions of the sample and reference arms to the image on the camera were adjusted to give the best fringe contrast. We employed a 1.5 × 1.5 cm polished quartz cuvette with a 1.5 cm width in the direction of the imaging probe pulse, but an optical path of only 2 mm in the glucose solution. The piezophotonic film was pressed between a glass window and the side of a quartz cuvette. This means that the photoacoustic wave generated in the film propagated through ~ 1.5 mm in quartz and 5 mm in solution before reaching the observation window. The Nd:YAG light was focused onto the piezophotonic film with lens L_pump_ (40-cm focal length; intentionally positioned ~ 35 cm from the film).

GUVs are very delicate and mobile microstructures. GUVs containing sucrose tend to deposit on the bottom of the cuvette filled with glucose solution. The propagation of ultrasound in quartz is much faster than in aqueous media and is not subject to acoustic reflection. Hence, deposited GUVs are subject to the fast and intense ultrasound propagating in the bottom of the cuvette, which biases their observation. Moreover, as soon as GUVs experience photoacoustic waves, they move in the field of observation toward the photoacoustic source. In order to keep the GUVs in the focus of the microscope and away from the bottom without perturbing their structure, we filled 2–3 mm of the cuvette with an aqueous gel made with gelatin and let the GUVs deposit on the gel. When the GUVs gently touch the gel, they stick to the gel. These stationary GUVs remain stable for hours. This allows for the imaging of virtually unperturbed GUVs in the focus of the microscope.

### Fluorescence image analysis

Fluorescence images were collected at 2 Hz. They were analysed with a combination of CellProfiler and Python scripts. A simple pipeline was developed in CellProfiler to identify primary objects and track them through the time-lapse images. The positional coordinates of the objects corresponding to the GUVs of interest were loaded along with the images into a python script which calculated the image intensity in the region corresponding to the GUV and baseline corrected that intensity based upon a region were no GUVs were present.

### Statistical analysis

All data are expressed as mean values or expressed as a percentage relative to control ± standard error of the mean. Statistical differences between groups were determined using Student’s t-test for independent samples and GraphPad Prism 5 software. Statistical significance was defined as *p* < 0.05. Two-tailed calculations were used. Ritgers-Peppas fitting of the fluorescence data was performed in R using a least squares fitting method.

## Supplementary Information

Supplementary Information.

ESM1 (WMV 3998 kb)

## Data Availability

Data described in this study will be available from the corresponding authors upon request.
