# Supplementary material for: Imaging of photoacoustic-mediated permeabilization of giant unilamellar vesicles (GUVs)
Source: Sci Rep. 2021 Feb 2;11:2775. doi: 10.1038/s41598-021-82140-4 (PMC7854711; doi:10.1038/s41598-021-82140-4)
Supplement: Supplementary file 1 — Supplementary Information. [file 41598_2021_82140_MOESM1_ESM.pdf]

Supplementary Information

Imaging of photoacoustic-mediated permeabilization of giant unilamellar vesicles (GUVs)

Diogo A. Pereira[1], Alexandre D. Silva[1], Patricia A. T. Martins[1], Ana P. Piedade[2], Dmitro Martynowych[3,4], David Veysset[4,5], Maria João Moreno[1], Carlos Serpa[1], Keith A. Nelson[3,4]*, Luis G. Arnaut[1]*

[1] Department of Chemistry, University of Coimbra, 3004-535 Coimbra, Portugal
[2] CEMMPRE-Department of Mechanical Engineering, University of Coimbra, 3030-788 Coimbra, Portugal
[3] Department of Chemistry, Massachusetts Institute of Technology, Cambridge, MA, 02139, USA
[4] Institute for Soldier Nanotechnology, Massachusetts Institute of Technology, Cambridge, MA, 02139, USA
[5]Hansen Experimental Physics Laboratory, Stanford University, Stanford, CA 94305, USA

* Correspondence and requests for materials should be addressed to K.A.N. (email: kanelson@mit.edu) or L.G.A. (email: lgarnaut@ci.uc.pt)

Supplementary Fig. 1. Absorption spectra of the films employed in this study.
Supplementary Fig. 2. Photoacoustic waves and their FFT measured with a 225 MHz contact transducer.
Supplementary Fig. 3. Flow cytometry of GUVs.
Supplementary Fig. 4. Fluorescence intensity of GUVs containing FITC-dextran quantified with ImageJ software.
Supplementary Fig. 5. Normalized area of GUVs measured by confocal microscopy.
Supplementary Fig. 6. Number of GUVs measured by flow cytometry.
Supplementary Fig. 7. PVA film after GUV formation by hydration of POPC containing 1 mol% Rho-DPPE
Supplementary Fig. 8. Best examples of release of GFP from GUVs.
Procedure to obtain absolute pressures from hydrophone measurements
Supplementary Fig. 9. Calibration of pressure waves generated by the CNT80-PDMS film after a 50 mJ/cm^2 pulse.
Supplementary Table 1. Dependence of the photoacoustic peak pressure on the distance from the source in water.
Supplementary Table 2. Relative composition of frequencies in the experimental photoacoustic wave of CNT80-PDMS film and calibration factors.
Supplementary Movie. Decrease of the fluorescence emitted by FITC-dextran contained in GUVs exposed to photoacoustic waves generated with a 10-Hz frequency, due to the release of FITC-dextran from the GUVs. The actual duration of the experiment was 15 min.

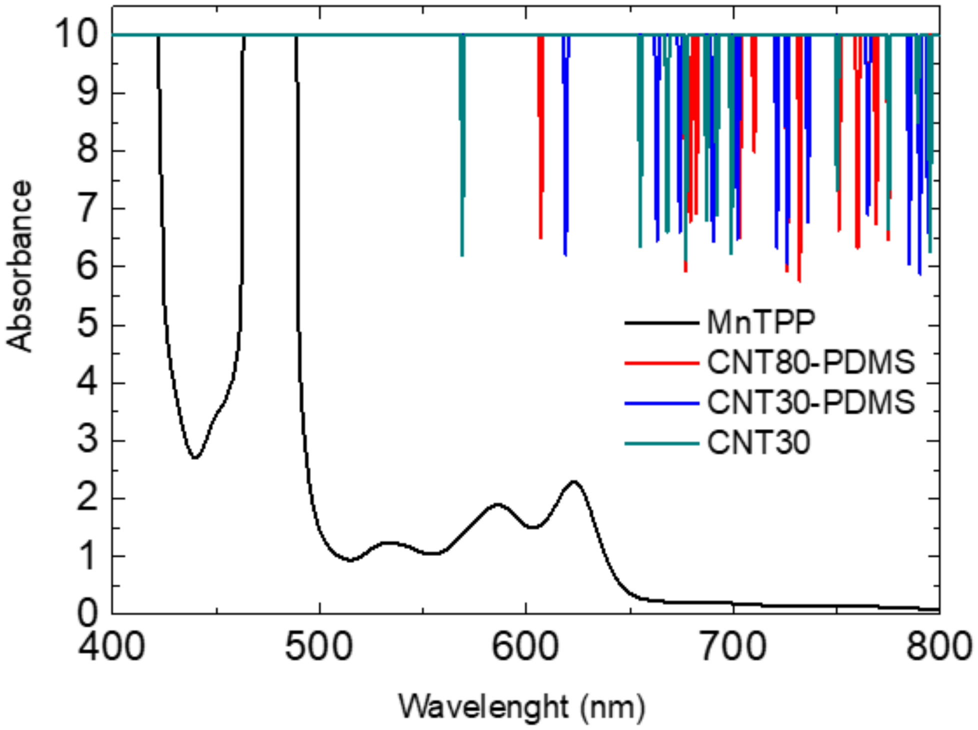

Supplementary Fig. 1. **Absorption spectra of the films employed in this study**. The lines in the 6 -10 absorbance range are artifacts due to the low level of light reaching the detector. The absorption is higher than 10 for all carbon nanotubes.

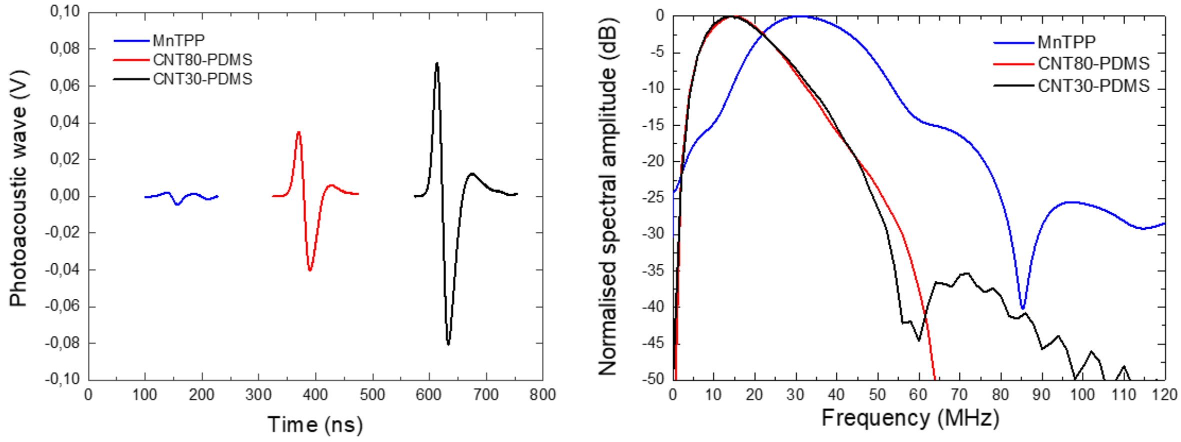

Supplementary Fig. 2. **Photoacoustic waves and their FFTs measured with a 225 MHz contact transducer**. The waves were generated with a pulsed laser at 532 nm wavelength and a fluence of 100 mJ/cm^2. The films were deposited on a glass slide and pressed against a back dielectric mirror with a thickness of 10 mm that reflects >99.9% of the light at 532 nm. The mirror was held firm against the transducer surface (Fig. 1). All parts were acoustically coupled with a thin layer of silicone. The waves in the left were arbitrarily separated in time to avoid their overlap. FFT was performed with OriginPro 2016.

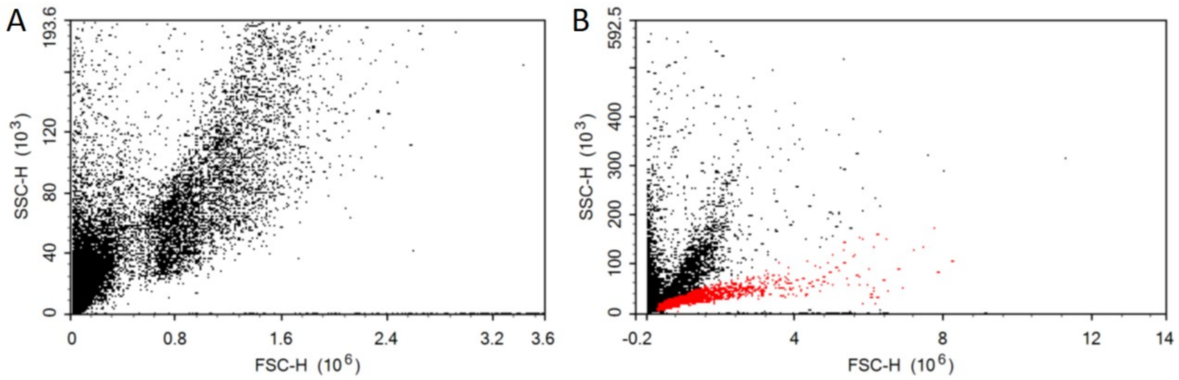

Supplementary Fig. 3. **Flow cytometry of GUVs**. The left panel presents the results from a solution of FITC-dextran 200 µM in sucrose 280 mM whereas the panel on the right reports the results from a similar solution with GUVs encapsulating FITC-dextran. The measured counts, represented in red, exclude the signal from FITC-dextran not encapsulated in the GUVs. This exclusion is based on forwarding and side-scatter characteristics. SSC-H: side-scatter height, FSC-H: forward-scatter height.

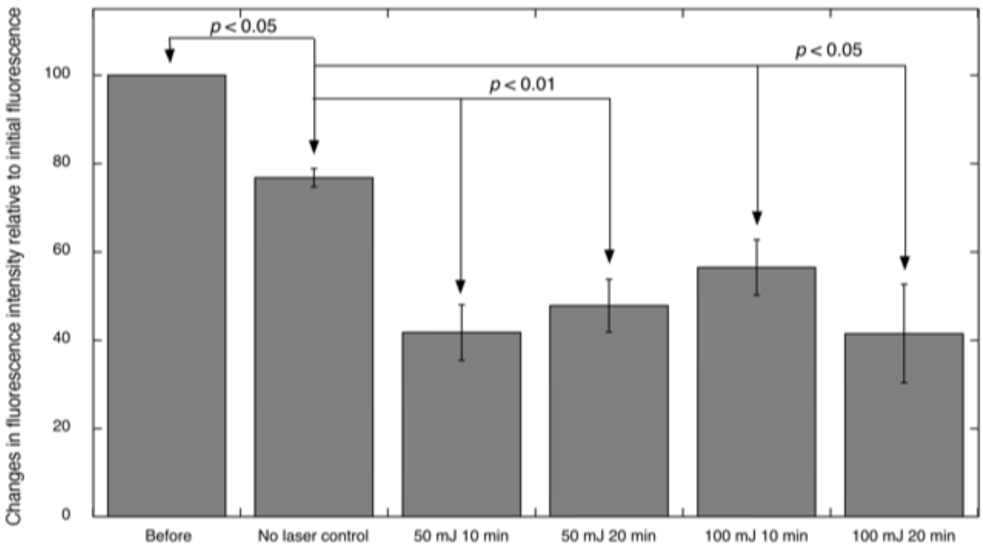

Supplementary Fig. 4. Decrease in relative fluorescence intensity of GUVs containing FITC-dextran quantified with ImageJ software. The data named "No laser control" refers to fluorescence decrease relative to initial fluorescence of samples (named "Before") that followed the same procedures as samples exposed to photoacoustic waves, but that were not exposed to such waves. Student's t test was applied to paired samples, comparing the same set of GUVs, prepared in one batch, before and after the exposure to photoacoustic waves or, in the case of the comparison between "Before" and "No laser control", the same set of GUVs before any procedures and after the procedures required for imaging and exposure to photoacoustic waves but with the laser off.

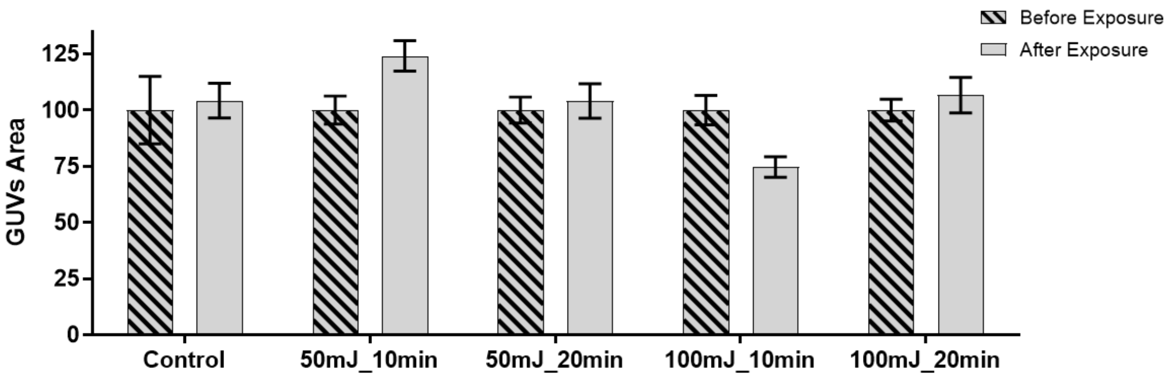

Supplementary Fig. 5. **Normalized area of GUVs measured by confocal microscopy**.

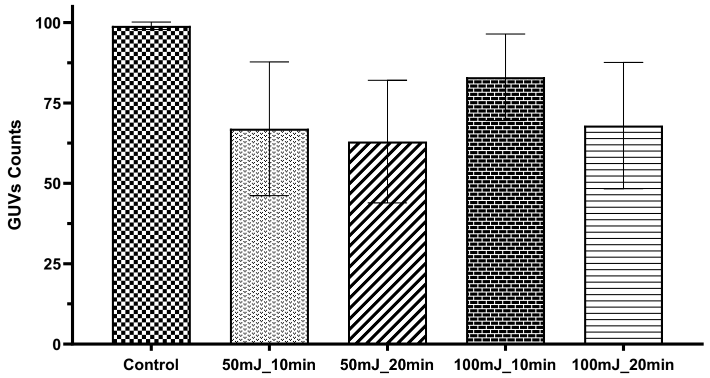

Supplementary Fig. 6. **Number of GUVs measured by flow cytometry**.

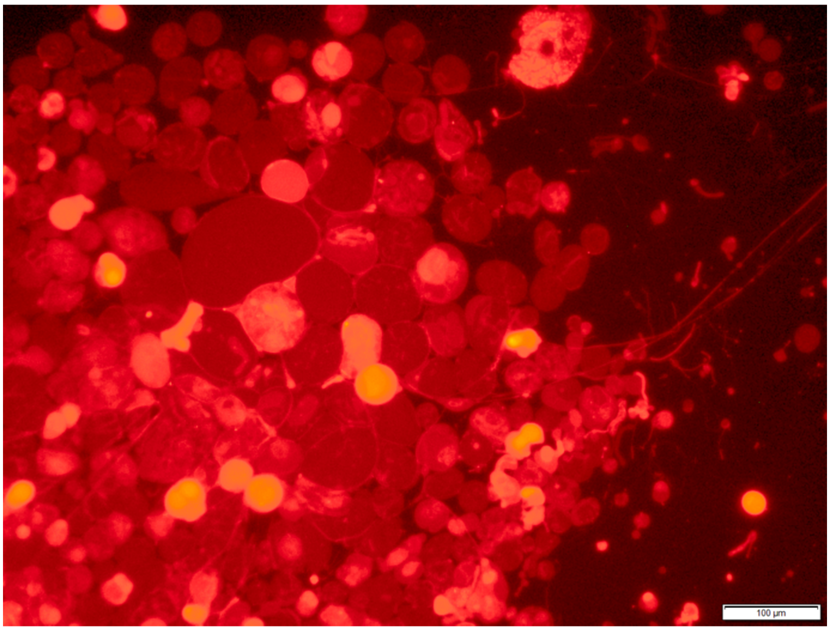

Supplementary Fig. 7. **PVA film after GUV formation by hydration of POPC containing 1 mol% Rho-DPPE**. Spherical bright fluorescent vesicles are clearly visible on top of the PVA film, which still contains some lipid film in the process of hydration/detachment. Scale bar 100 μm.

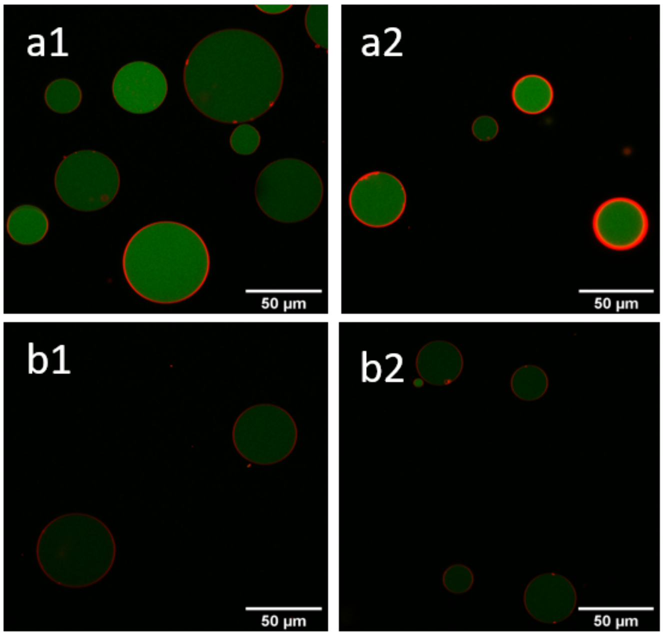

Supplementary Fig. 8. Best examples of release of GFP from GUVs. **a,** Images of GUVs before exposure to photoacoustic waves. **b,** Images of GUVs after exposure to photoacoustic waves generated with laser pulses at 100 mJ/cm^2 for 10 min illustrating some of the most significant decreases in fluorescence intensity associated with the release of GFP.

Procedure to obtain absolute pressures from hydrophone measurements

Pressure waves measured with a 0.2 mm 30 MHz needle hydrophone (Precision Acoustics model NH0200) in a water pool at 20 °C and registered with DPO7254 Tektronix digital oscilloscope (2.5 GHz bandwidth) were converted to absolute pressures following the instructions of the manufacturer. The procedure to obtain absolute pressures is illustrated below with the data collected for the CNT80-PDMS film.

The measured pressure wave is shown in the left of Supplementary Fig. 8. Fast Fourier transformation (FFT) of this wave using OriginPro 2016 with a sampling interval of 2.5x10^{11} led to the frequency distribution shown in the centre of Supplementary Fig. 8. Next, we obtained the contribution of each frequency, with a step of 1 MHz from 0 to 30 MHz, to the total distribution, as shown in Supplementary Table 1. This table also includes the calibration factors for the various frequencies, which allows us to calculate the final calibration factor, 73.3mV/MPa. This is the calibration factor that converts the measured pressure wave with absolute pressures shown in the right of Supplementary Fig. 8.

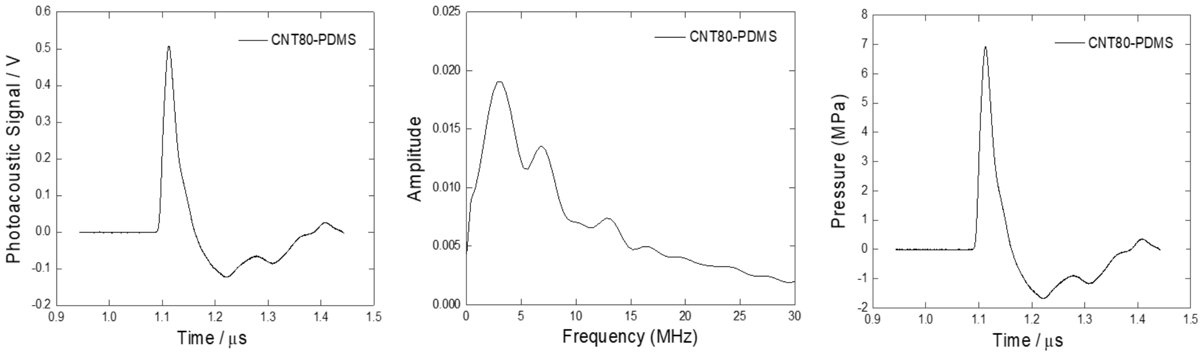

Supplementary Fig. 9. **Calibration of pressure waves generated by the CNT80-PDMS film after a 50 mJ/cm^2 pump pulse**. (Left) Experimental pressure wave as measured with a 30 MHz needle hydrophone. (Centre) Frequency distribution obtained by FFT of the wave in the left. (Right) Absolute pressures obtained by the multiplication of the wave in the left by the calibration factor.

Supplementary Table 1. Dependence of the photoacoustic peak pressure on the distance from the source in water, measured with a hydrophone, for a laser beam diameter of 3 mm using two laser fluences.

50 mJ cm^{-2}		100 mJ cm^{-2}	
Distance / mm	p_{max} / MPa	Distance / mm	p_{max} / MPa
3	1.80	3	2.44
5	1.41	5	2.02
7	1.24	7	1.75
10	1.03	10	1.45

Supplementary Table 2. Relative composition of frequencies in the experimental photoacoustic wave of CNT80-PDMS film and calibration factors.

MHz	Contribution	%	MHz	mV/MPa	Calibration (mV/MPa)
1	0.01084	5.22	1	52	2.71
2	0.01631	7.85	2	73	5.73
3	0.01903	9.16	3	82	7.51
4	0.0166	7.99	4	89	7.11
5	0.01226	5.90	5	77	4.55
6	0.01229	5.92	6	76	4.50
7	0.01341	6.46	7	82	5.29
8	0.01074	5.17	8	77	3.98
9	0.00766	3.69	9	66	2.43
10	0.00705	3.39	10	59	2.00
11	0.00667	3.21	11	64	2.06
12	0.00685	3.30	12	77	2.54
13	0.00735	3.54	13	75	2.65
14	0.00619	2.98	14	71	2.12
15	0.00481	2.32	15	68	1.57
16	0.00492	2.37	16	70	1.66
17	0.00481	2.32	17	71	1.64
18	0.00421	2.03	18	70	1.42
19	0.00409	1.97	19	71	1.40
20	0.00396	1.91	20	72	1.37
21	0.00358	1.72	21	73	1.26
22	0.00335	1.61	22	75	1.21
23	0.00328	1.58	23	74	1.17
24	0.00324	1.56	24	72	1.12
25	0.00294	1.42	25	67	0.95
26	0.00251	1.21	26	64	0.77
27	0.00246	1.18	27	64	0.76
28	0.00233	1.12	28	62	0.70
29	0.00198	0.95	29	60	0.57
30	0.00197	0.95	30	59	0.56
Total	0.20769	100.00	**Value (mV/MPa)**		73.33
